# Does Prophylactic Negative-Pressure Wound Therapy Prevent Surgical Site Infection After Laparotomy? A Systematic Review and Meta-analysis of Randomized Controlled trials

**DOI:** 10.1007/s00268-023-06908-7

**Published:** 2023-01-19

**Authors:** Jeremy Meyer, Elin Roos, Richard Justin Davies, Nicolas Christian Buchs, Frédéric Ris, Christian Toso

**Affiliations:** 1 Division of Digestive Surgery, University Hospitals of Geneva, Rue Gabrielle-Perret-Gentil 4, 1211 Geneva 14, Switzerland; 2 Unit of Surgical Research, Medical School, University of Geneva, Geneva, Switzerland; 3 Cambridge Colorectal Unit, Addenbrooke’s Hospital, Cambridge University Hospitals NHS Foundation Trust, Cambridge, UK; 4 Department of Global Public Health, Karolinska Institutet, Solna, Sweden

## Abstract

**Background:**

Prophylactic negative-pressure wound therapy (pNPWT) may prevent surgical site infection (SSI) after laparotomy, but existing meta-analyses pooling only high-quality evidence have failed to confirm this effect. Recently, several randomized controlled trials (RCTs) have been published. We performed an updated systematic review and meta-analysis to determine if pNPWT reduces the incidence of SSI after laparotomy.

**Methods:**

MEDLINE, Embase, CENTRAL and Web of Science were searched on the 25.08.2021 for RCTs reporting on the incidence of SSI in patients who underwent laparotomy with and without pNPWT. The systematic review was compliant with the AMSTAR2 recommendation and registered into PROSPERO. Risk ratios (RR) for SSI in patients with pNPWT, and risk difference (RD) between control and pNPWT patients, were obtained using random effects models. Heterogeneity was quantified using the *I*^2^ value, and investigated using subgroup analyses, funnel plots and bubble plots. Risk of bias of included RCTs was assessed using the RoB2 tool.

**Results:**

Eleven RCTs were included, representing 973 patients who received pNPWT and 970 patients who received standard wound dressing. Pooled RR and RD between patients with and without pNPWT were of, respectively, 0.665 (95% CI 0.49–0.91, *I*^2^: 38.7%, *p* = 0.0098) and −0.07 (95% CI −0.12 to −0.03, *I*^2^: 53.6%, *p* = 0.0018), therefore demonstrating that pNPWT decreases the incidence of SSI after laparotomy. Investigation of source of heterogeneity identified a potential small-study effect.

**Conclusion:**

The protective effect of pNPWT against SSI after laparotomy is confirmed by high-quality pooled evidence.

**Supplementary Information:**

The online version contains supplementary material available at 10.1007/s00268-023-06908-7.

## Introduction

Surgical site infection (SS) affects 12.3% of patients undergoing abdominal surgery worldwide, an incidence that increases with the contamination level of the surgical wound [1]. SSI leads to deleterious consequences for healthcare systems, in terms of costs (estimated to range between 20′785 USD [2] and 49′449 USD [3] per episode of SSI) and prevalence of antibiotic resistance (which is estimated to be of 21.6% [1]), but also for patients, who endure prolonged length of stay, and increased 30-day incidences of reintervention and mortality [1, 3–5].

To reduce the incidence of SSI, the World Health Organization recommends applying prophylactic negative-pressure wound therapy (pNPWT) [6]. pNPWT consists of an airtight wound dressing material, connected to an aspiration pump that applies a controlled level of sub-atmospheric pressure onto a closed wound, in order to prevent wound-related complications and to accelerate healing. Postulated mechanisms include aspiration of the wound exudate, prevention of retrograde bacterial contamination of the wound by the skin flora, reduction of tissue oedema, tightening of the wound edges and improvement of wound healing by stimulation of neovascularization [7]. Several dedicated commercial devices have been developed for this purpose, such as the PREVENA incision management system (KCI, Acelity), the PICO single use negative-pressure wound therapy system (Smith and Nephew), the VSD Vacuum Sealing Device (Wuhan VSD Medical Science and Technology) and the NPseal (Guard Medical).

The effect of pNPWT on the prevention of wound-related complications was considered to decrease the incidence of SSI after surgery according to the latest Cochrane systematic review (with moderate certainty level of evidence) [8]. Similarly, encouraging results were reported in surgical subspecialties by systematic reviews and meta-analyses of randomized controlled trials (RCTs), notably in vascular surgery (groin incision) [9] and in orthopedic surgery [10].

In abdominal surgery, a recent systematic review and meta-analysis of RCTs and observational studies showed that pNPWT reduced the incidence of SSI by 12 percentage points [risk difference (RD): −12%, 95% CI −17 to −8%, *I*^2^: 54%, *p* <0.00001] and was protective against the occurrence of SSI with a relative risk (RR) of 0.53 (95% CI 0.40–0.71, *I*^2^: 56%, *p* <0.0001). However, this effect became borderline when performing subgroup analysis pooling only RCTs (RD: −12%, 95% CI −22 to −1%, *I*^2^: 69%, *p* = 0.03; odds ratio: 0.47, 95% CI 0.22–1.00, *I*^2^: 67%, *p* = 0.05) [11]. Subsequently, systematic reviews and meta-analyses of RCTs pooling together patients undergoing laparotomy and Cesarean section reported a significant effect of pNPWT in reducing the incidence of SSI [12, 13]. However, this effect was not confirmed by meta-analyses of RCTs including only patients who underwent laparotomy [14–16], which all pooled the same five RCTs [17–21]. Considering that the effect of the technique was found to be more pronounced in studies pooling patients with an incidence of SSI ≥ 20% in the control arm [11] (who are patients at higher risk for SSI), the absence of effect demonstrated by meta-analyses of RCTs so far was probably due to the low number of pooled patients (590 patients) and because of heterogeneity in terms of pNPWT interventions (due to different commercial devices, different subatmospheric pressures applied, different durations of therapy). Therefore, recommendation was made to wait for more RCTs to be released, notably RCTs including patients at higher risk for SSI [22].

Since the publication of the latest meta-analyses of RCTs in the field [16], several RCTs have been released, allowing an update of the current evidence and to potentially overcome the limitations of previous meta-analyses and reach a more definitive conclusion.

The objective of the present systematic review and meta-analysis was therefore to determine if pNPWT decreases the incidence of SSI after laparotomy, pooling only high-quality evidence.

## Materials and methods

The systematic review complied with the PRISMA (Preferred Reporting Items for Systematic Reviews and Meta-Analyses) [23] (Table S1) and AMSTAR 2 (Assessing the methodological quality of systematic reviews) statements, and was registered into the international prospective register of systematic reviews PROSPERO (CRD42021275532). MEDLINE, Embase, Web of Science and CENTRAL were searched on the 25.08.2021 for RCTs reporting the incidence of SSI in patients who underwent laparotomy with and without pNPWT (Tables S2 and S3). RCTs comparing pNPWT to conventional wound dressing (without subatmospheric pressure) in patients who underwent laparotomy were considered for inclusion. Abstracts, conference papers, studies not in English, studies including patients who had other incisions than laparotomy (such as Caesarian-section and groin incision), studies including patients who did not undergo abdominal closure (such as patients with open abdomen) and/or studies not reporting the incidence of SSI at 1 month in both groups were excluded. Two independent reviewers (JM, ER) performed the screening of eligible articles and the data extraction, using the software Covidence (Covidence systematic review software, Veritas Health Innovation, Melbourne, Australia). In case of disagreement, consensus was reached with a third reviewer (FR). Characteristics of included studies [first authors, country, year of publication, study design, number of centers involved, study period, population, setting, type of access to the abdominal cavity, number of patients, intervention (type of pNPWT device, pressure applied and duration of therapy), control, definition of SSI as outcome, timepoint(s) used for the SSI outcome, number of patients per allocated group and number of patients per group who experienced SSI] were extracted from included studies. Risk difference (RD) between control and pNPWT patients and risk ratios (RR) for SSI in patients with pNPWT were obtained using models with random effects [24]. The number needed to treat (NNT) was calculated as 1/(−RD). Heterogeneity was quantified using the *I*^2^ value, and was investigated using subgroup analyses (per RCT sample size, per type of pNPWT device and per quality of RCT), cumulative meta-analysis and meta-regression. Meta-regression was performed using the magnitude of effect of pNPWT [in terms of log(RR)] as the dependent variable and the sample size (number of patients) of included RCTs as the independent variable. Cumulative meta-analysis explored the trend in the magnitude of effect of pNPWT as a function of sample size, by progressively adding studies to the pooled analysis.

Publication bias was assessed visually using funnel plot and corrected using the trim-and-fill methods. Small-study effect was looked for using the Egger test [25]. Risk of bias of included RCTs was assessed using the revised Cochrane risk of bias tool for randomized trials (RoB2 tool) [26]. The software STATA 17 was used for the analyses (StataCorp. 2021. Stata Statistical Software: Release 17. College Station, TX: StataCorp LLC).

## Results

### Inclusion process

**Fig. 1:**
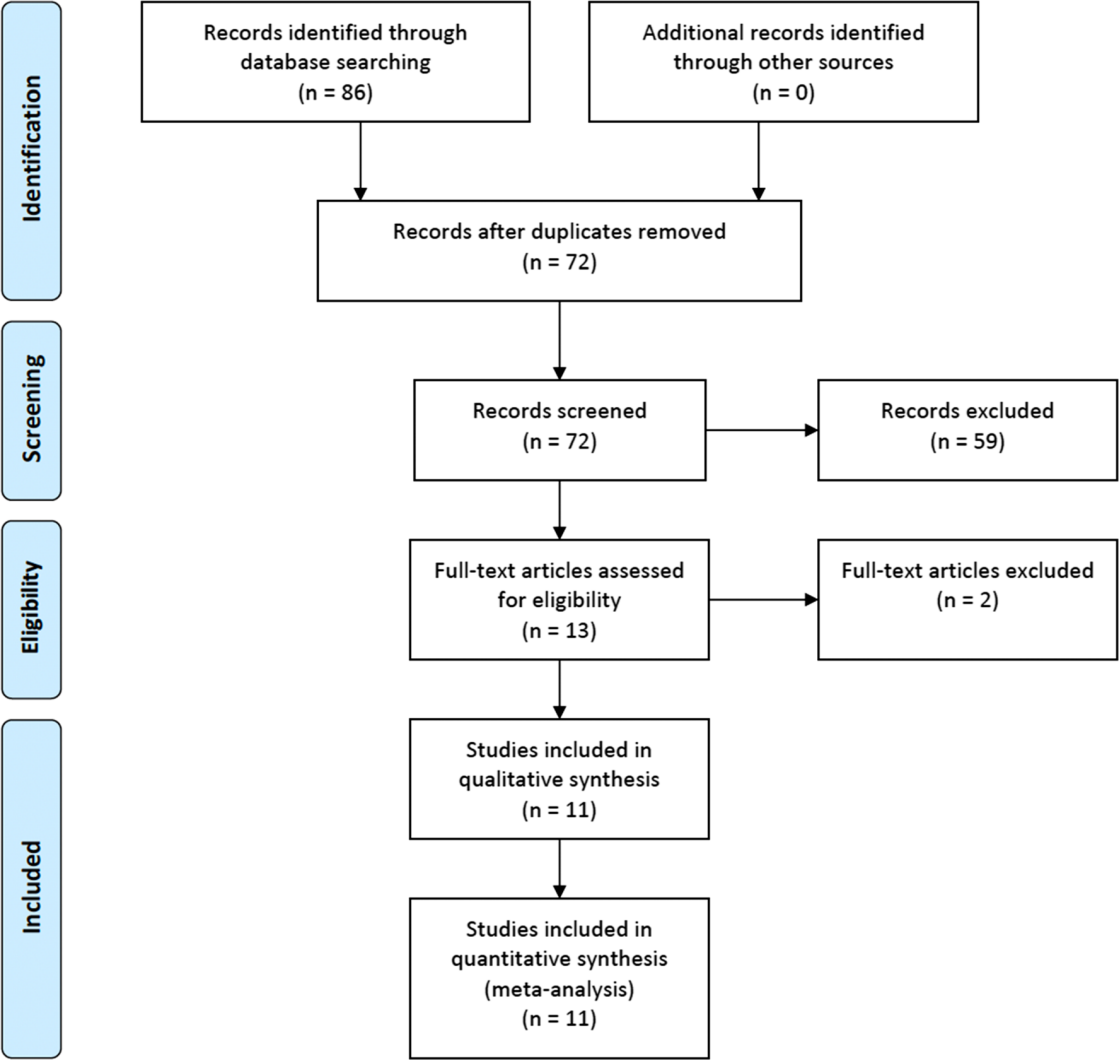
PRISMA flowchart of the inclusion process

Eighty-six publications were identified from database screening. Fourteen duplicates were removed. Of the 72 publications remaining, 59 were excluded after title and abstract screening and two after full text screening [27, 28], leaving 11 studies for inclusion [17–21, 28–34] (Fig. 1). The RCT by Javed et al. was included even though the epidermis was not closed in the intervention group [17].

### Characteristics of included studies

There was a total of 1943 patients in included RCTs, with 973 of the patients being allocated to pNPWT and 970 to conventional wound dressing. The number of patients per RCT ranged between 40 [33] and 505 [32]. Studies were published recently, one in 2016 [21], two in 2017 [18, 20], two in 2019 [17, 19], three in 2020 [30, 31, 33] and three in 2021 [29, 32, 34]. Four RCTs were performed in the USA [17, 21, 32, 33], two in Australia [31, 34], two in Spain [29, 30], one in Canada [19], one in Ireland [20] and one in China [18]. Four RCTs included patients who underwent abdominal/digestive surgery [18, 20, 21, 31], two included patients who had hepato-biliary surgery [17, 33], two included patients who underwent colorectal surgery [19, 29], one included patients who had incisional hernia repair [30], one included patients who underwent laparotomy for gynaecologic surgery [32], and one included patients who had general surgery [34]. Four RCTs considered patients who underwent elective and emergency surgical interventions [20, 29, 31, 34], and six RCTs only included elective patients [17, 19, 21, 30, 32, 33]. Five RCTs assessed the Prevena device [17, 19, 29, 32, 34], four RCTs evaluated the PICO device [20, 30, 31, 33], and one RCT tested the VSD device [18]. One RCT did not report the specifications of the tested device [21], and the authors did not respond to contact attempts. Pressure applied varied from −80 mmHg (which is the standard pressure for the PICO device) [20, 30, 31, 33] to −125 mmHg [17–19, 29, 32, 34]. Duration of therapy ranged from 3 [18] to 7 days [29, 30, 33]. Detailed characteristics of included studies are reported in Table 1.

**Table 1:** Characteristics of included studies

Authors	Year	Country	Design	Mono/multicenter	Period	Population	Setting	Access	Patients, *n*	Intervention	Control	Definition of SSI	Timepoint
Arellano et al.	2021	Spain	RCT	Multicenter	02.2016–08.2016	Colorectal surgery	Elective and emergency surgery	Laparotomy	148	Prevena (7 days)	Conventional wound dressing changed every 24 h	CDC: superficial incisional + deep incisional + organ space	7, 15 and 30 days
Bueno-Lledo et al.	2020	Spain	RCT	Monocenter	05.2017–01.2020	Incisional hernia repair	Elective surgery	Laparotomy	146	PICO (−80 mmHg, 7 days)	Conventional wound dressing	CDC: superficial incisional + deep incisional + organ space	30 days
Flynn et al.	2020	Australia	RCT	Monocenter	03.2015–09.2017	Digestive surgery	Elective and emergency surgery	Laparotomy	188	PICO (−80 mmHg, 7 days or less if discharge)	Conventional wound dressing	VICNISS: superficial incisional + deep incisional + organ space	7 days and more
Javed et al.	2019	USA	RCT	Monocenter	01.2017–02.2018	Pancreaticoduodenectomy	Elective surgery	Laparotomy	123	Prevena (−125 mmHg, 5 days)	Conventional wound dressing	CDC: superficial incisional + deep incisional	30 days
Leitao et al.	2021	USA	RCT	Multicenter	03.2016–08.2019	Gynecologic surgery	Elective surgery	Laparotomy	505	Prevena (−125 mmHg, 7 days or less if discharge)	Conventional wound dressing	“Wound infection”	30 days
Li et al.	2017	China	RCT	Monocenter	05.2015–12.2015	Abdominal surgery	–	Laparotomy	71	VSD (−125 mmHg, 3 days)	Conventional wound dressing	CDC: superficial incisional + deep incisional	30 days
Murphy et al.	2019	Canada	RCT	Monocenter	01.2015–02.2017	Colorectal surgery	Elective surgery	Laparotomy	284	Prevena (−125 mmHg, 5 days or less if discharge)	Conventional wound dressing	CDC: superficial incisional	30 days
O’Leary et al.	2017	Ireland	RCT	Monocenter	02.2013–04.2016	Abdominal surgery	Elective and emergency surgery	Laparotomy	49	PICO (−80 mmHg, 4 days)	Conventional wound dressing	CDC: superficial incisional + deep incisional	30 days
O’Neill et al.	2020	USA	RCT	Monocenter	10.2017–09.2018	Hepato-pancreatic surgery	Elective surgery	Laparotomy	40	PICO (− 80 mmHg, 7 days)	Conventional wound dressing	CDC: superficial incisional + deep incisional + organ space	3, 7, 15 and 30 days
Re et al.	2021	Australia	RCT	Multicenter	2015–2019	General surgery	Elective and emergency surgery	Laparotomy	124	Prevena (−125 mmHg, 5–7 days)	Conventional wound dressing	Superficial SSI	5–7 days, 30 days
Shen et al.	2016	USA	RCT	Monocenter	06.2012–06.2016	Abdominal oncologic surgery	Elective surgery	Laparotomy	265	Unknown (−125 mmHg, 4 days)	Conventional wound dressing	CDC: superficial incisional + deep incisional	30 days

*RCT* randomized controlled trial, *CDC* center for diseases control and *VICNISS* victorian healthcare associated infection surveillance system

### Quality assessment of included studies

According to the RoB2 tool, all included RCTs were at high risk of bias or with concerns, notably for domains 3 and 4 (Table S4).

### Investigation of the postulated protective effect of pNPWT against SSI

**Fig. 2:**
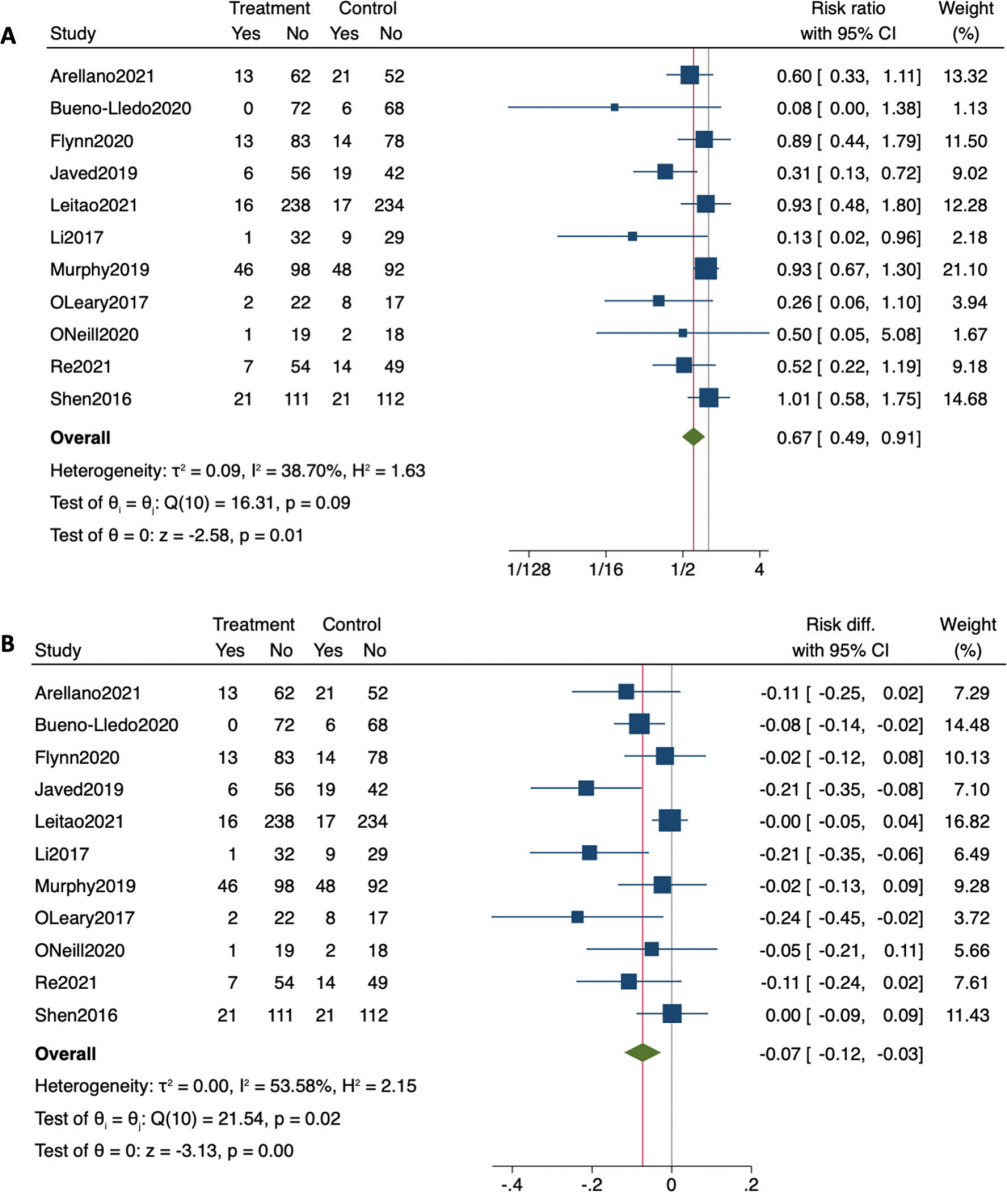
Meta-analysis assessing the risk of SSI in patients with and without pNPWT. **a** Pooled relative risk (RR). **b** Pooled risk difference (RD). Each horizontal bar summarizes a study. The bars represent 95% confidence intervals. The gray squares inform on each of the studies’ weight in the meta-analysis. The diamond in the lower part of the graph depicts the pooled estimate along with 95% confidence intervals. Events (yes) = patients with surgical site infection (SSI). The gray line indicates no effect (RR = 0). The red line indicates the pooled effect

Meta-analysis of the eleven included RCTs, representing 1943 patients, found that pNPWT was significantly protective against the occurrence of SSI within 30 days of the index laparotomy, with a pooled RR of 0.665 (95% CI 0.488–0.906, *I*^2^: 38.70%, *p* = 0.0098) (Table 2; Fig. 2a, b). Further, pNPWT led to significant reduction in the incidence of SSI in patients with pNPWT when compared to patients without pNPWT by 7.3 percentage units (95% CI 2.7–11.9%, *I*^2^: 53.58%, *p* = 0.0018) (Table 2; Fig. 2b). This RD corresponds to a NNT of 13.70 patients (95% CI 8.40–37.03).

**Table 2:** Pooled relative risk for SSI between pNPWT and control patients

Subgroup analyses		Studies, *n*	RR (95% CI)	*I*^2^ (%)	*P* value	*P* value for difference between groups
All studies		11	0.665 (0.488–0.906)	38.70	0.0098	–
Sample size	≤100 patients	3	0.245 (0.086–0.699)	0	0.0086	0.048
	>100 patients	8	0.737 (0.549–0.988)	36.02	0.0410	
	≤150 patients	7	0.429 (0.290–0.635)	0	<0.0001	0.001
	>150 patients	4	0.940 (0.736–1.201)	0	0.6225	
Device	PREVENA	5	0.676 (0.464–0.983)	46.46	0.0406	0.502
	PICO	4	0.481 (0.192–1.205)	32.42	0.1183	

Relative risk (RR) was obtained using models with random effects. Heterogeneity was assessed using the *Q*-test and quantified using the *I*^2^ value. Risk of bias was assessed by using the Cochrane Collaboration’s tool for assessing risk of bias for randomized controlled trials (RoB2). Subgroups analyses were performed according to the sample size of included studies, the type of pNPWT commercial device used and the methodological quality of included studies

*RR* relative risk and *CI* confidence interval

### Exploration of heterogeneity

**Fig. 3:**
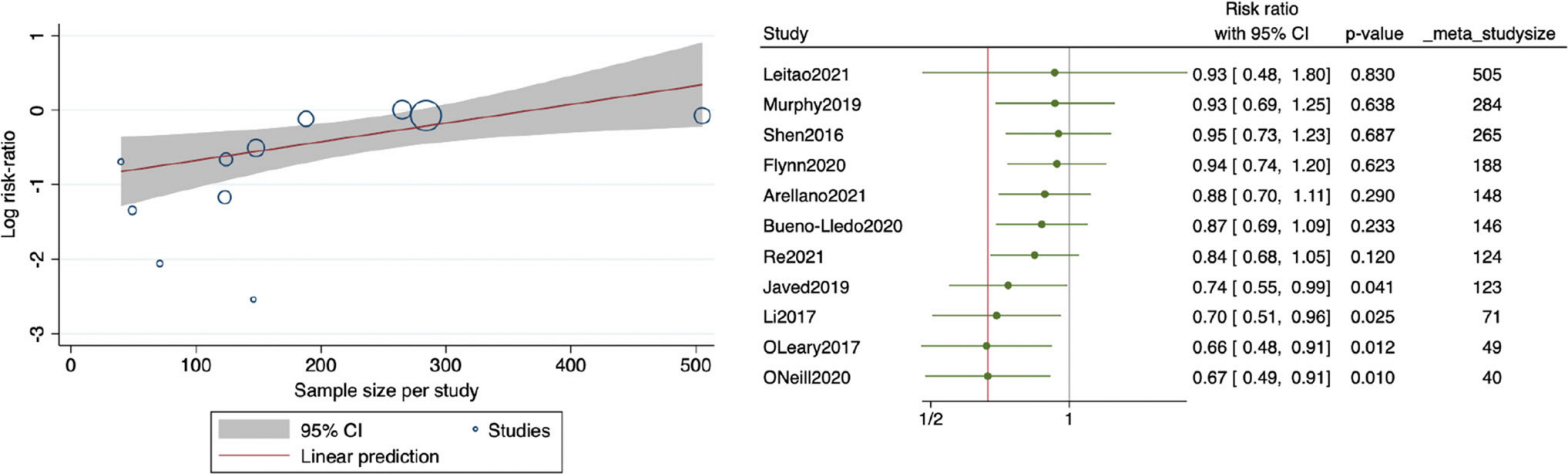
Meta-regression and cumulative meta-analysis. Left: meta-regression assessing the risk of SSI in patients [in terms of log(RR)] with and without pNPWT plotted against the sample sizes of included studies. Each circle summarizes a study. The red line depicts the pooled estimate along with 95% confidence intervals in gray. Negative values indicate a protective effect against the occurrence of SSI. Right: cumulative meta-analysis by decreasing sample size. Included studies are added one by one to the pooled analysis. The gray line indicates no effect (RR = 0). The red line indicates the pooled effect

Heterogeneity was investigated using subgroup analyses and meta-regression. When pooling only RCTs including patients who received the Prevena device, pNPWT conserved its beneficial effect in preventing SSI (5 RCTs, RR: 0.676, 95% CI 0.464–0.983, *I*^2^: 46.46%, *p* = 0.0406). However, when pooling only RCTs which evaluated the PICO device, statistical significance was lost (4 RCTs, RR: 0.481, 95% CI 0.192–1.205, *I*^2^: 23.42%, *p* = 0.1183). The effect of pNPWT seemed to be more pronounced in RCTs with smaller sample sizes. For instance, RCTs including 100 patients and less reported a strong protective effect conferred by the technique (RR: 0.245, 95% CI 0.086–0.699, *I*^2^: 0%, *p* = 0.0086). This effect was significantly less important (*p* = 0.048 between subgroups) when pooling only RCTs including more than 100 patients, but remained significant (RR: 0.737, 95% CI 0.549–0.988, *I*^2^: 36.02%, *p* = 0.0410). Moreover, setting a sample size threshold at 150 patients reduced heterogeneity to 0% within subgroups (less or more than 150 patients) and confirmed that the effect of the technique was more pronounced in small studies (*p* = 0.001 between subgroups). When pooling RCTs including 150 patients and less, pNPWT was protective against SSI (RR: 0.429, 95% CI 0.290–0.635, *I*^2^: 0%, *p* <0.0001). However, statistical significance was lost when pooling RCTs including more than 150 patients (RR: 0.940, 95% CI 0.736–1.201, *I*^2^: 0%, *p* = 0.6225) (Tables 2 and 3). Considering that sample size was suspected to constitute a cause for heterogeneity, meta-regression was performed and established the existence of an association between the magnitude of effect of pNPWT [in terms of log(RR)] and the sample size of included RCTs (coefficient: 0.00251, 95% CI 0.00005–0.00449, *p* = 0.0130) (Fig. 3a). Cumulative meta-analysis finally explored the trend in the magnitude of effect of pNPWT as a function of sample size and visually showed that RCTs including fewer patients tended to pull the pooled RR toward a lower RR (and therefore a greater effect of the technique) (Fig. 3b).

**Table 3:** Pooled risk difference for SSI between pNPWT and control patients

Subgroup analyses		Studies, *n*	RD (95% CI)	*I*^2^ (%)	*P* value	*P* value for difference between groups
All studies		11	−0.073 (−0.119– −0.027)	53.58	0.0018	–
Sample size	≤100 patients	3	−0.158 (−0.271– −0.045)	24.06	0.0062	0.096
	>100 patients	8	−0.054 (−0.099– −0.010)	49.21	0.0267	
	≤150 patients	7	−0.120 (−0.167– −0.073)	7.92	<0.0001	<0.001
	>150 patients	4	−0.007 (−0.041–0.027)	0.00	0.6861	
Device	PREVENA	5	−0.078 (−0.155– −0.001)	63.68	0.0462	0.887
	PICO	4	−0.071 (−0.130– −0.012)	16.58	0.0181	

Risk difference (RD) was obtained using models with random effects. Heterogeneity was assessed using the *Q*-test and quantified using the *I*^2^ value. Risk of bias was assessed by using the Cochrane Collaboration’s tool for assessing risk of bias for randomized controlled trials (RoB2). Subgroups analyses were performed according to the sample size of included studies, the type of pNPWT commercial device used and the methodological quality of included studies

*RD* risk difference and *CI* confidence interval

### Exploration of publication bias and small-study effect

**Fig. 4:**
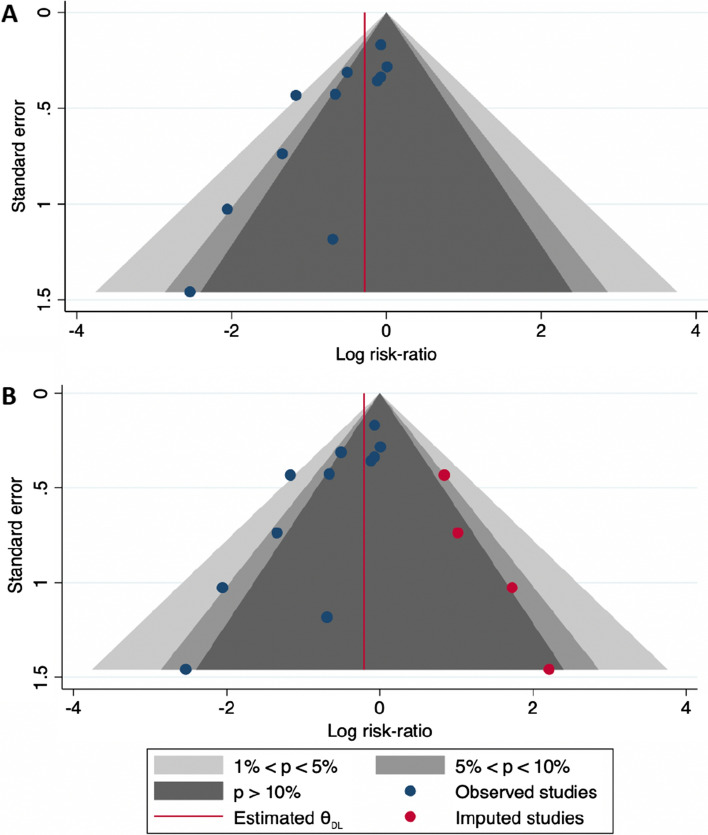
Funnel plots for the investigation of a potential publication bias. **a** Funnel plot for the investigation of a potential publication bias, reporting the log(relative risk). Gray full circles represent the studies included in the meta-analysis. The red line indicates the pooled effect. **b** Funnel plot with imputed missing studies. The red dots represent the studies suspected to be unpublished (as identified by the trim-and-fill approach). The red line indicates the pooled effect

Publication bias was explored using funnel plot. Visual analysis of funnel plot revealed the potential absence of RCTs in favor of conventional wound dressing [bottom right part of the funnel plot (Fig. 4a)]. Potential small-studies effect, as suggested by subgroups analyses, was investigated using the Egger regression-based test. The estimated slope *β*1 was −1.88 with a standard error of 0.590, giving a test statistic of *z* = −3.18 and a *p* value of 0.0015, confirming funnel plot asymmetry and suggesting a publication bias. After properly accounting for heterogeneity due to sample size through the inclusion of sample size in the calculation (as moderator), the estimated slope *β*1 was −1.50 with a standard error of 0.657, giving a test statistic of *z* = 2.28 and a *p* value of 0.0229. This suggests that other factors than small-study effect may explain the publication bias. Thereafter, four potential missing studies were imputed into the calculation using the trim-and-fill methods. This led to a reduced pNPWT effect, with a RR increasing from 0.665 to 0.812, with a wider 95% CI that overlapped the value of 1 (RR: 0.812, 95% CI 0.567–1.165) (Table 4; Fig. 4b).

**Table 4:** Pooled measures of the intervention’s effect corrected for a potential publication bias

Trimm and fill analysis	Studies, *n*	Log RR (95% CI)	RR (95% CI)
Observed studies	11	−0.408 (−0.717– −0.098)	0.665 (0.488–0.906)
**Observed + imputed studies**	15	−0.208 (−0.568–0.153)	0.812 (0.567–1.165)

To investigate for a potential publication bias, we inspected the symmetry of funnel plots. We applied the trim-and-fill method to identify studies potentially missing because of a publication bias and to assess the pooled intervention’s effect corrected for a potential publication bias. Imputed studies are the number of included studies plus the number of studies identified with the trim-and-fill approach that should be added for the funnel plot to become symmetric. Relative risk (RR) corrected for publication bias was reported

*RR* relative risk and *CI* confidence interval

## Discussion

Meta-analysis of eleven RCTs confirms the findings made by observational studies, that pNPWT protects against the occurrence of SSI within 30 days of index laparotomy (RR: 0.665, *p* = 0.0098). Of note, use of the technique decreased the incidence of SSI by 7.3 percentages units. This latest finding may be of importance for healthcare systems, considering that SSI was previously reported to have an incidence of 26.6% after laparotomy [11] and a cost as high as 49,449 USD [3].

The RD determined using meta-analysis of RCTs is lower than the RD reported by previous meta-analyses of both RCTs and observational studies [11]. This suggests a potential publication bias against negative observational studies, therefore increasing the postulated effect of the intervention in these meta-analyses. Nevertheless, the beneficial effect of pNPWT on the incidence of SSI is now confirmed by high-quality evidence. This updated RD may be useful for sample size calculation for future RCTs in the field and/or for potential cost-benefit studies.

The RR and RD reported had moderate heterogeneity of, respectively, 38.70–53.58%, which was investigated by subgroups analyses and meta-regression. Subgroups analyses identified the type of commercial device used in the intervention group as one cause for heterogeneity. For instance, pooled RR values were statistically different between RCTs using the Prevena device and RCTs using the PICO device. Moreover, statistical significance was lost when pooling only RCTs which used the PICO device, although better RR value was found. In addition, the effect of pNPWT was more pronounced in RCTs with smaller sample sizes, and lost significance when considering only RCTs including more than 150 patients. Meta-regression confirmed that the magnitude of effect of pNPWT depended on the sample size of included RCTs. Cumulative meta-analysis provided the same conclusion and showed that small RCTs pulled the pooled effect to the left of the forest plot (better effect of the technique). Therefore, the observed discrepancy between RCTs with small and larger sample sizes suggests a small-study effect [35]. Visual analysis of the funnel plot of included RCTs identified missing studies with small sample size and in favor of the control group. The Egger test confirmed the existence of a publication bias. Correcting for the suspected missing studies using the trim-and-fill methods reduced the effect of the intervention. Therefore, it is possible that RCTs with small sample size were not published due to negative results. When interpreting these data, it should also be beared in mind that previous meta-analysis showed that the effect of the intervention was higher in populations with higher risk of SSI (>20% in the control group) [36]. Therefore, differences between studies with small sample sizes and studies with larger sample sizes may also be explained by different types of populations.

All included RCTs were considered to be at high risk of bias (or with some concerns) according to the RoB2 tool. Of note, no RCT adequately addressed management of the missing outcome data and/or failed reporting the primary outcome for all randomized patients, which gave poor evaluation for domain 3. Moreover, in the majority of RCTs, the outcome assessor was not blinded, which can lead to a significant risk of reporting bias. This was reflected by sub-optimal notations for domain 4. We believe that blinding of the primary outcome (incidence of SSI) assessor is of crucial importance and may be achieved by removing the pNPWT device or the conventional wound dressing 24 h before wound assessment (to avoid suction or glue marks on the skin) for early outcome measurement, and by not detailing the type of wound dressing applied in the operative note.

The NNT is of importance to assess the cost-effectiveness of the intervention. The NNT to avoid one occurrence of SSI was previously reported to be of 9 patients (95% CI 6–13 patients) by a systematic review and meta-analysis of observational studies and RCTs [11]. In the present meta-analysis of RCTs, the NNT was slightly higher and of 13.70 patients (95% CI 8.40–37.03). We believe that the cost-effectiveness of pNPWT should be carefully evaluated by future studies before drawing definitive recommendations regarding its routine use in clinical practice.

The main strength of the present systematic review and meta-analysis is that is shows a beneficial effect of pNPWT in patients who underwent laparotomy, by including more RCTs than previous analysis that reached opposite conclusion [14–16]. Its second strength is the thorough analyses performed, which allowed to identify potential publication bias and small-study effect.

The main limitation of the present study is the low number of publications identified by the literature search strategy. However, the latter was designed to be specific and nevertheless identified all RCTs included in the previous systematic reviews in the field [14–16]. Moreover, most existing meta-analyses pooled the same five RCTs [17–21] and did not find a significant effect of the intervention, whereas we included 11 RCTs and reached different conclusion. The second limitation of the present meta-analysis is the identification of a small-study effect and potential publication biais, which may limit its conclusion. And its third limitation is the language restriction applied during the systematic review, which may have led to ignore potential eligible RCTs in other languages.

In conclusion, this systematic review and meta-analysis of RCTs confirmed the beneficial effect of pNPWT in preventing the occurrence of SSI after laparotomy. Of note, pNPWT allowed significant reduction in the incidence of SSI in patients with pNPWT by 7.3 percentage points. However, this result has to be mitigated by potential publication bias and small-study effect, and the cost-effectiveness of the technique remains to be investigated.

## Supplementary Information

Below is the link to the electronic supplementary material.

Supplementary file1 (DOC 66 KB)

Supplementary file2 (DOCX 14 KB)

Supplementary file3 (DOCX 16 KB)

Supplementary file4 (DOCX 18 KB)

## Abbreviations

NNT: Number needed to treat
OR: Odds ratio
pNPWT: Prophylactic negative-pressure wound therapy
RCT: Randomized controlled trial
RD: Risk difference
SSI: Surgical site infection

## References

[1] GlobalSurg C (2018). Surgical site infection after gastrointestinal surgery in high-income, middle-income, and low-income countries: a prospective, international, multicentre cohort study. Lancet Infect Dis.

[2] Zimlichman E, Henderson D, Tamir O (2013). Health care-associated infections: a meta-analysis of costs and financial impact on the US health care system. JAMA Intern Med.

[3] Badia JM, Casey AL, Petrosillo N (2017). Impact of surgical site infection on healthcare costs and patient outcomes: a systematic review in six European countries. J Hosp Infect.

[4] Urban JA (2006). Cost analysis of surgical site infections. Surg Infect (Larchmt).

[5] Mahmoud NN, Turpin RS, Yang G (2009). Impact of surgical site infections on length of stay and costs in selected colorectal procedures. Surg Infect (Larchmt).

[6] Allegranzi B, Zayed B, Bischoff P (2016). New WHO recommendations on intraoperative and postoperative measures for surgical site infection prevention: an evidence-based global perspective. Lancet Infect Dis.

[7] Borgquist O, Ingemansson R, Malmsjo M (2011). Individualizing the use of negative pressure wound therapy for optimal wound healing: a focused review of the literature. Ostomy Wound Manage.

[8] Norman G, Goh EL, Dumville JC (2020). Negative pressure wound therapy for surgical wounds healing by primary closure. Cochrane Database Syst Rev.

[9] Gombert A, Dillavou E, D'Agostino R (2020). A systematic review and meta-analysis of randomized controlled trials for the reduction of surgical site infection in closed incision management versus standard of care dressings over closed vascular groin incisions. Vascular.

[10] Ailaney N, Johns WL, Golladay GJ (2021). Closed incision negative pressure wound therapy for elective hip and knee arthroplasty: a systematic review and meta-analysis of randomized controlled trials. J Arthroplast.

[11] Meyer J, Roos E, Abbassi Z (2020). Prophylactic negative-pressure wound therapy prevents surgical site infection in abdominal surgery: an updated systematic review and meta-analysis of randomized controlled trials and observational studies. Clin Infect Dis.

[12] Gong S, Yang J, Lu T (2021). Incisional negative pressure wound therapy for clean-contaminated wounds in abdominal surgery: a systematic review and meta-analysis of randomized controlled trials. Expert Rev Gastroenterol Hepatol.

[13] Wells CI, Ratnayake CBB, Perrin J (2019). Prophylactic negative pressure wound therapy in closed abdominal incisions: a meta-analysis of randomised controlled trials. World J Surg.

[14] Zwanenburg PR, Tol BT, Obdeijn MC (2020). Meta-analysis, meta-regression, and GRADE assessment of randomized and nonrandomized studies of incisional negative pressure wound therapy versus control dressings for the prevention of postoperative wound complications. Ann Surg.

[15] Kuper TM, Murphy PB, Kaur B (2020). Prophylactic negative pressure wound therapy for closed laparotomy incisions: a meta-analysis of randomized controlled trials. Ann Surg.

[16] Boland PA, Kelly ME, Donlon NE (2021). Prophylactic negative pressure wound therapy for closed laparotomy wounds: a systematic review and meta-analysis of randomised controlled trials. Ir J Med Sci.

[17] Javed AA, Teinor J, Wright M (2019). Negative pressure wound therapy for surgical-site infections: a randomized trial. Ann Surg.

[18] Li PY, Yang D, Liu D (2017). Reducing surgical site infection with negative-pressure wound therapy after open abdominal surgery: a prospective randomized controlled study. Scand J Surg.

[19] Murphy PB, Knowles S, Chadi SA (2019). Negative pressure wound therapy use to decrease surgical nosocomial events in colorectal resections (NEPTUNE): a randomized controlled trial. Ann Surg.

[20] O'Leary DP, Peirce C, Anglim B (2017). Prophylactic negative pressure dressing use in closed laparotomy wounds following abdominal operations: a randomized, controlled, open-label trial: the P.I.C.O Trial. Ann Surg.

[21] Shen P, Blackham AU, Lewis S (2017). Phase II randomized trial of negative-pressure wound therapy to decrease surgical site infection in patients undergoing laparotomy for gastrointestinal, pancreatic, and peritoneal surface malignancies. J Am Coll Surg.

[22] Roos E, Toso C, Meyer J Comment on: meta-analysis, meta-regression, and GRADE assessment of randomized and nonrandomized studies of incisional negative pressure wound therapy versus control dressings for the prevention of postoperative wound complications. Ann Surg Accepted for publication 10.1097/SLA.0000000000003841 32068558

[23] Page MJ, McKenzie JE, Bossuyt PM (2021). The PRISMA 2020 statement: an updated guideline for reporting systematic reviews. Int J Surg.

[24] DerSimonian R, Laird N (1986). Meta-analysis in clinical trials. Control Clin Trials.

[25] Lin L, Chu H (2018). Quantifying publication bias in meta-analysis. Biometrics.

[26] Sterne JAC, Savovic J, Page MJ (2019). RoB 2: a revised tool for assessing risk of bias in randomised trials. BMJ.

[27] Gok MA, Kafadar MT, Yegen SF (2019). Comparison of negative-pressure incision management system in wound dehiscence: a prospective, randomized, observational study. J Med Life.

[28] Kuncewitch MP, Blackham AU, Clark CJ (2019). Effect of negative pressure wound therapy on wound complications post-pancreatectomy. Am Surg.

[29] Arellano ML, Serrano CB, Guedea M (2021). Surgical wound complications after colorectal surgery with single-use negative-pressure wound therapy versus surgical dressing over closed incisions: a randomized controlled trial. Adv Skin Wound Care.

[30] Bueno-Lledo J, Franco-Bernal A, Garcia-Voz-Mediano MT (2021). Prophylactic single-use negative pressure dressing in closed surgical wounds after incisional hernia repair: a randomized, controlled trial. Ann Surg.

[31] Flynn J, Choy A, Leavy K (2020). Negative pressure dressings (PICO(TM)) on laparotomy wounds do not reduce risk of surgical site infection. Surg Infect (Larchmt).

[32] Leitao MM, Zhou QC, Schiavone MB (2021). Prophylactic negative pressure wound therapy after laparotomy for gynecologic surgery: a randomized controlled trial. Obstet Gynecol.

[33] O'Neill CH, Martin RCG (2020). Negative-pressure wound therapy does not reduce superficial SSI in pancreatectomy and hepatectomy procedures. J Surg Oncol.

[34] Di Re AM, Wright D, Toh JWT (2021). Surgical wound infection prevention using topical negative pressure therapy on closed abdominal incisions—the ‘SWIPE IT’ randomized clinical trial. J Hosp Infect.

[35] Sterne JA, Gavaghan D, Egger M (2000). Publication and related bias in meta-analysis. J Clin Epidemiol.

[36] Meyer J, Roos E, Abbassi Z (2021). Prophylactic negative-pressure wound therapy prevents surgical site infection in abdominal surgery: an updated systematic review and meta-analysis of randomized controlled trials and observational studies. Clin Infect Dis.

