# Supplementary material for: Does Prophylactic Negative-Pressure Wound Therapy Prevent Surgical Site Infection After Laparotomy? A Systematic Review and Meta-analysis of Randomized Controlled trials
Source: World J Surg. 2023 Jan 19;47(6):1464–74. doi: 10.1007/s00268-023-06908-7 (PMC10156868; doi:10.1007/s00268-023-06908-7)
Supplement: Supplementary file 2 — Supplementary file2 (DOCX 14 KB) [file 268_2023_6908_MOESM2_ESM.docx]

| Population | Patients undergoing laparotomy |
| --- | --- |
| Intervention | Prophylactic negative-pressure wound therapy |
| Control | Conventional wound dressing |
| Outcome | Incidence of surgical site infection |
| Study design | Meta-analysis of randomized controlled trials |
