# Supplementary material for: Does Prophylactic Negative-Pressure Wound Therapy Prevent Surgical Site Infection After Laparotomy? A Systematic Review and Meta-analysis of Randomized Controlled trials
Source: World J Surg. 2023 Jan 19;47(6):1464–74. doi: 10.1007/s00268-023-06908-7 (PMC10156868; doi:10.1007/s00268-023-06908-7)
Supplement: Supplementary file 3 — Supplementary file3 (DOCX 16 KB) [file 268_2023_6908_MOESM3_ESM.docx]

| **Database** | **Search build** |
| --- | --- |
| MEDLINE | ((surgical site infection[Title/Abstract]) OR SSI[Title/Abstract]) OR (infection, surgical wound[MeSH Terms]) OR (wound infection, surgical[MeSH Terms]))  AND  ((Negative-pressure therapy[Title/Abstract]) OR (Negative pressure therapy[Title/Abstract]) OR (Negative-pressure wound therapy[Title/Abstract]) OR (Negative pressure wound therapy[Title/Abstract]) OR (Prophylactic closed-incision negative-pressure wound therapy[Title/Abstract]) OR (Prophylactic closed-incision negative pressure wound therapy[Title/Abstract]) OR (NPT[Title/Abstract]) OR (NPWT[Title/Abstract]) OR (pNPT[Title/Abstract]) OR (pNPWT[Title/Abstract]))  AND  ((laparotomy[Title/Abstract]) OR (open abdominal surgery[Title/Abstract]) OR (abdominal surgery[Title/Abstract]) OR (abdominal incision[Title/Abstract]) OR (open colorectal surgery[Title/Abstract]) OR (pancreaticoduodenectomy[Title/Abstract]) OR (hepatectomy[Title/Abstract]) OR (abdominal wall reconstruction[Title/Abstract]) OR (peritonitis[Title/Abstract]) OR (acute care surgery[Title/Abstract]) OR (closed incision[Title/Abstract]) OR (closed abdominal wound[Title/Abstract]) OR (midline incision[Title/Abstract]))  AND  ((randomized controlled trial[MeSH Terms]) OR (clinical trials, randomized[MeSH Terms]) OR (controlled clinical trials, randomized[MeSH Terms]) OR (RCT[Title/Abstract]) OR (random*[Title/Abstract])) |
| EMBASE | ('surgical site infection':ti,ab,kw OR 'SSI':ti,ab,kw OR 'surgical infection'/exp/mj)  AND  ('negative-pressure therapy':ti,ab,kw OR 'negative pressure therapy':ti,ab,kw OR 'negative-pressure wound therapy':ti,ab,kw OR 'negative pressure wound therapy':ti,ab,kw OR 'NPWT':ti,ab,kw)  AND  ('laparotomy':ti,ab,kw OR 'open abdominal surgery':ti,ab,kw OR 'abdominal surgery':ti,ab,kw OR 'abdominal incision':ti,ab,kw OR 'abdominal wound':ti,ab,kw OR 'midline incision':ti,ab,kw)  AND  ('randomized controlled trial'/exp/mj OR 'randomized controlled trial':ti,ab,kw OR 'RCT':ti,ab,kw) |
| WEB OF SCIENCE | (TI=(surgical site infection))  AND  (TI=(negative-pressure therapy) OR TI=(negative pressure therapy) OR TI=(negative-pressure wound therapy) OR TI=(negative pressure wound therapy) OR TI=(NPWT))  AND  (TI=(laparotomy) OR TI=(abdominal surgery) OR TI=(abdominal incision) OR TI=(abdominal wound) OR TI=(midline wound) OR TI=(open))  AND  (TI=(randomized controlled trial) OR TI=(RCT)) |
| CENTRAL | ('surgical site infection':ti,ab,kw)*  AND  ('negative-pressure therapy':ti,ab,kw)*  AND  ('laparotomy':ti,ab,kw OR 'abdominal surgery':ti,ab,kw OR 'abdominal surgery':ti,ab,kw 'midline incision':ti,ab,kw)  AND  ('randomized controlled trial'/exp/mj OR 'randomized controlled trial':ti,ab,kw OR 'RCT':ti,ab,kw)  *With word variations |
