# Supplementary material for: Does Prophylactic Negative-Pressure Wound Therapy Prevent Surgical Site Infection After Laparotomy? A Systematic Review and Meta-analysis of Randomized Controlled trials
Source: World J Surg. 2023 Jan 19;47(6):1464–74. doi: 10.1007/s00268-023-06908-7 (PMC10156868; doi:10.1007/s00268-023-06908-7)
Supplement: Supplementary file 4 — Supplementary file4 (DOCX 18 KB) [file 268_2023_6908_MOESM4_ESM.docx]

| Study | Domain 1 | Domain 2 | Domain 3 | Domain 4 | Domain 5 | Conclusion |
| --- | --- | --- | --- | --- | --- | --- |
| Arellano et al. 2021 | NI-NI-PN | Y-Y-NI-NA-NA-NI-NI | Y-NA-NA-NA | PN-PN-NI-Y-PY | PN-PN-PN | High risk of bias |
| Bueno-Lledo et al. 2020 | Y-Y-PN | Y-Y-PN-NA-NA-NI-PN | N-N-PY-PN | PN-PN-Y-PY-PY | Y-PN-PN | High risk of bias |
| Flynn et al. 2020 | NI-Y-N | Y-Y-PY-PN-NA-PN-PY | N-N-PY-PY | PN-PN-Y-Y-PY | PN-PN-PN | High risk of bias |
| Javed et al. 2019 | Y-Y-N | Y-Y-PN-NA-NA-Y-NA | N-N-PY-PY | PN-PN-Y-PY-PY | NI-PN-PN | High risk of bias |
| Leitao et al. 2021 | Y-Y-N | Y-Y-PN-NA-NA-PY-NA | N-N-PY-PY | PN-PN-Y-PY-PY | PY-PN-PN | High risk of bias |
| Li et al. 2017 | Y-Y-N | Y-Y-PN-NA-NA-PY-NA | Y-NA-NA-NA | PN-PN-N-NA-NA | PN-PN-PN | Some concerns |
| Murphy et al. 2019 | Y-PY-PN | Y-Y-PY-PY-N-Y-NA | N-N-Y-Y | PN-PN-N-NA-NA | Y-N-N | High risk of bias |
| O’Leary et al. 2017 | Y-PY-N | Y-Y-PN-NA-NA-PN-PN | N-N-PY-PY | PN-PN-Y-PY-PY | Y-N-N | High risk of bias |
| O’Neill et al. 2020 | NI-PN-N | Y-Y-PN-NA-NA-PY-NA | Y-NA-NA-NA | PN-PN-PY-PY-PY | PN-PY-PY | High risk of bias |
| Re et al. 2021 | Y-Y-N | Y-Y-PN-NA-NA-PN-PY | N-N-PY-PY | PN-PN-Y-PY-PY | Y-N-N | High risk of bias |
| Shen et al. 2016 | NI-PN-N | Y-Y-PY-PY-Y-N-PY | N-N-Y-PY | PN-PN-Y-PY-PY | Y-PN-PN | High risk of bias |
